# Letter to the Editor of the Southern Medical and Surgical Journal

**Published:** 1845-03

**Authors:** 


We offer no apology to our readers, for inserting the following interesting letter, from our young and talented friend, Dr. Cumming, now of Amoy, (China.) This is an answer to a request to contribute to the pages of the Medical Journals of our country; and we hope, in a few months, to be in the regular receipt of valuable articles and information directly from abroad.

*Southern Med. and Surg. Journal.*

"In your letter, you request me to send home accounts of our medical operations. Up to this time there has been so little of order and method in my practice, that I have had few opportunities of observing cases long enough and well enough for description. Of the history of the cases, there is often little or nothing known by the patients. They seem to forget the dates and peculiarities of their disorders with the greatest facility. But as we learn more of the language, this difficulty will be diminished, as we may do much towards refreshing their memories by pertinent questions. As yet, all description must be most general. The most common of all the disorders is gastralgia (generally complicated with pyrosis)—of 388 new cases received during February and March, there were 68 of this disease, 13 of simple indigestion, 9 of simple pyrosis, making 90 affections of the stomach. Of coughs (principally bronchitis) 56, asthma 15, rheumatism 17, pains (from falls, &c.) 18, of affections of the skin 20, and miscellaneous medical cases 23. Of keratitis 32, conjunctivitis 25, blepharitis 18, opacity of cornea 14, trichiasis 6, iritis 3, staphyloma of iris 3, miscellaneous affections of the eye 7, (of which one of melanosis)—eye cases 108; syphilis 17, other affections of the genital organs 5, otitis 3, ulcers 8, miscellaneous medical cases 8. Of all these diseases, the acute inflammations of the eye and the affections of the stomach are most frequently cured. For the former we cup, purge, blister and anoint. I have recently been much pleased with an ointment of sulphate of copper—I use from 8 gr. to 16 gr. per ounce of lard. For gastralgias, &c. we have almost a specific in a preparation of pepper 5 parts, and rhubarb 6 parts; we make 133 pills of an ounce of this mixture, and gave six pills daily, 2 an hour before each meal; it has done admirably thus far, (nearly two years.) For the cough, we use ipecac. or tartar emetic pills, with some success (12 gr. of the former or 3 of the latter, in twelve doses daily.) Many cases of asthma are much relieved by belladonna and ipecac. pills. For rheumatisms we blister and give Dover's powders. For syphilis, corrosive sublimate pills 1-6 gr. each, beginning with two a day and going on to ten. In cases of opacity of cornea, we blow into the eye a mixture of sugar candy and red precipitate, finely powdered—this is done from two to six times daily. In these we are quite successful. Of hydroceles, we see a great many—I punctured two to-day, but our patients are generally satisfied with having it emptied, go away very much rejoiced and never come back. We have quite a number of miscellaneous surgical cases, such as whitlows, abscesses, wounds, (especially among the sailors,) bruises, &c. &c.

"I suppose that you have heard that Dr. Hepburn, of the Presbyterian Board, came here in November. He is fast picking up the language, and is a good deal interested in medical matters. We rented two houses in Amoy about the end of the year, and I came over the 19th January. Since the opening of our dispensary here, we have many more patients than before. Since the beginning of February, Dr. H. and I have had more than 560 new cases, averaging 10 daily—they are also of a more interesting kind than formerly, there being a far larger proportion of acute cases. Our dispensary consists of a front room 42 by 21, in which the patients are seated, and a back room 18 by 21, in which are our medicines and in which we carry on our operations. We have two assistants (Chinese servants) who can cup, spread blisters, &c., make pills and help us in many ways. I am desirous of getting three or four youths trained as regular assistants; with these, we could accomplish far more than at present. My teacher thinks of learning the business. Of medicines we have had a pretty good supply, and we expect that Mr. Boone will make permanent arrangements on this point. We are even now looking out for a stock just arrived from the United States. We have opened a hospital also, principally for cases of cataract. We have room for 50 patients, but have now only eight. If we succeed in our first operations, for cataract, I think we shall have multitudes of cases. What we need is skill, and if we acquire that, we may do a great deal of good. In time, I have no doubt that we shall be able to send home some interesting articles, but it will take considerable additions to my knowledge both of medicine and Chinese, before such memoirs can have much value.

"Our missionary medical body in China is increasing in number—A Dr. McCarter, of New-York, has recently arrived, sent out by the Presbyterian Board, with a printer. We learn from home, that Mr. Boone hopes to bring out a number of new missionaries—they will be welcome, for they are much needed. Dr. McGowan, of the Baptist Board, expects to settle at Ningpo—he was there during the winter, and had many patients. As soon as we can have access to the neighboring cities, we shall have an immense field for medical practice; and I think it likely that we should be tolerated where no one else would. Within forty miles from Amoy, there are probably more than three millions of people—How fine a field for medical enterprise! Amoy might be made the central station, from which medicines, &c. could be forwarded from other places. In a few years there will be ample employment for scores of physicians. And if we hope to raise up men among the Chinese to practice the healing art, we cannot expect that three or four teachers, having their hands full of work, will be able to do much. If those Christians who complain that they can find nothing to do as physicians at home, would come hither, their complaints would soon cease. And for men anxious to learn, here is a fine opportunity. If we had the funds for a large hospital, we could easily keep it full. By feeding the patients, we could keep them as long as we desired, and by judicious selection we could soon beat any hospital in Europe, for we have a population around us, and an absence of competition which would draw hither all, of medical importance, for many leagues in the interior, so that La Charite and L'Hotel Dieu of Paris, would be completely eclipsed. May that day come."

